# How Patients With Schizophrenia Use the Internet: Qualitative Study

**DOI:** 10.2196/jmir.1550

**Published:** 2010-12-19

**Authors:** Beate Schrank, Ingrid Sibitz, Annemarie Unger, Michaela Amering

**Affiliations:** ^1^Department of Psychiatry and PsychotherapyMedical University of ViennaViennaAustria

**Keywords:** Schizophrenia, psychosis, Internet, attitudes, behaviors

## Abstract

**Background:**

The Internet is an important source of health information for people with psychiatric conditions. Little is known about the way patients with schizophrenia use the Internet when it comes to issues related to their illness. Data on their specific needs, difficulties, and the consequences related to Internet use are lacking.

**Objective:**

Our objective was to investigate the nature and subjective consequences of health-related Internet use among patients with schizophrenia.

**Methods:**

In all, 26 individual semistructured interviews were conducted and analyzed qualitatively in groups of 4 until theoretical saturation was achieved.

**Results:**

Study results suggest that the Internet is an influential source of illness-related information for patients with schizophrenia. Many aspects of their behavior around the Internet resemble those of individuals not afflicted by mental illness. Importantly, problems specific to patients with schizophrenia were stimulus overflow, an inability to deal with the abundance of information, difficulties with concentration, lack of energy, paranoid ideas, symptom provocation, and the need to distance themselves from illness-related topics as part of the recovery process. Internet information was subjectively perceived as having the potential to significantly change patients’ attitudes toward medication and their relationships with doctors.

**Conclusions:**

These findings provide insight into how individuals with schizophrenia handle illness-related Internet information. The data could contribute to the continuous development of Internet-based interventions and offer novel approaches to optimizing traditional treatment options.

## Introduction

Private use of the Internet as a source of information is increasing worldwide. Today, in Austria, 70% of households have access to the Internet, and 67% of the general population regularly uses the Web [1-2]. Likewise, the Internet is of growing importance specifically as a source of health information [3]. How it is actually used and the importance attributed to online health information varies among different patient groups, such as cancer, gynecology, or general practice patients [4]. Due to its anonymity and easy access, however, the Internet is a particularly important source of information and opportunity for peer exchange for those suffering from chronic or stigmatizing conditions [5-6].

A representative survey of the general population in Great Britain found that 18% of all Internet users access information on mental health issues [7], and, as revealed in a survey among psychiatric outpatients in Switzerland, about 68% of those with various mental health problems use the Internet as a source of information related to their diagnosis [8]. A further study of people with a major mental illness in the United States found that about one-third use the Internet and about half of these access health information online [9]. Despite the fact that online health information is of varying quality and readability [10-12], the Internet may exert considerable influence on its users by enhancing coping strategies, empowerment, and self-efficacy; by decreasing the feelings of anxiety and isolation; and by affecting the doctor-patient relationship as well as health-related behaviors and decisions, as has been shown in qualitative and quantitative studies with participants suffering from both common and severe mental illness [3-4,13-15].

Due to their often-marked interpersonal difficulties, people with schizotypal personality disorder have been found to be especially likely to use the Internet, with a particular interest in social interaction on the Web [16]. Similar considerations apply to schizophrenia, given the stigma and the interpersonal communication problems frequently associated with this illness. The resulting social anxiety and retreat may make the Internet a particularly important realm of possibility for this group of patients. However, symptoms of schizophrenia such as attention deficit or delusional interpretations may become a barrier to Internet use, especially since websites containing information on schizophrenia are usually difficult to read, as found in a recent study on patient information for schizophrenia on the Web [11].

These complex preconditions indicate that Internet use related to issues concerning schizophrenia may be associated with certain difficulties, needs, and consequences specific to patients suffering from this illness. However, currently available knowledge on the effects of Internet use on patients has been largely generated in medical fields other than mental health. Psychiatric research in this area has so far focused mainly on depression and anxiety disorders [17-24] or mixed psychiatric patient groups [25]. Some of these studies using a qualitative approach have found, for example, that people with mood disorders increasingly turn to the Internet to make health care decisions, but are also often merely looking for emotional support, sympathy, social companionship, and help with getting through the day [17-18]. At the same time, the Internet offers a stage for pretenders seeking attention by faking illnesses such as depression, and this may have profound negative consequences for patients using online interaction in a spirit of honesty [17]. Cross-sectional quantitative research suggests that user-selectable peer support may actually aggravate psychological burden and thus have the potential to trigger a downward depressive spiral [19]. By the same token, longitudinal quantitative research found that using the Internet for health purposes may be associated with increased depression, attributable to increased rumination, unnecessary alarm, or overattention to health problems and self-selected online health resources [20]. On the other hand, various Web-based interventions, ranging in their focus from self-help to structured professionally led therapies, have been shown to reduce symptoms of depression and anxiety [21-23]. Overall, however, the methodological quality of such intervention studies is low, and high-quality randomized controlled trials are needed to inform the practice of consumers, practitioners, and policy makers [24].

When it comes to severe mental illnesses such as schizophrenia, the impact of the broad availability of illness-related information from the Internet on patients with schizophrenia remains almost entirely unknown. Hence, as a first step, this study aims to uncover the complex and differentiated experiences and insights of people with schizophrenia and the potential subjectively experienced consequences of Internet use on illness-related attitudes, behaviors, and relationships with doctors.

## Methods

### Sample

Participants were eligible for inclusion if the following criteria applied: (1) diagnosis of schizophrenia or schizoaffective disorder according to *the International Classification of Diseases, Tenth Revision* (*ICD-10*) [26], (2) age 18 to 65, (3) being stable enough to participate in the interview, and (4) current or past use of the Internet. Purposive sampling was used to maximize the likelihood of obtaining a broad range of views. Hence, the target group consisted of people of different age ranges, sociodemographic backgrounds, and varying levels of Internet use overall and for illness-related information or interaction in particular. Participants were recruited from the outpatient department and the day clinic of the Department of Psychiatry and Psychotherapy at the Medical University of Vienna, from community psychiatrists, and Promente, a low threshold community mental health organization that also confirmed the patients’ diagnoses. The study was approved by the responsible ethics committee, and all participants gave written informed consent for participation before the interview. A consultant psychiatrist was available during and after the interviews in case a participant might feel burdened or distressed as a result of the interview. None of the participants requested any intervention.

### Qualitative Interviews

A semistructured interview style was employed because previous research in other fields suggested a number of areas of interest. Semistructured interviews allowed those areas to be covered while at the same time providing the flexibility to explore emerging themes and individual issues in detail. Accordingly, an interview guide was generated from a literature review, with the initial topics including the extent of Internet use as a means to gain information about the illness; illness-related interaction with others on the Internet; reasons for and against using the Internet for these purposes and consequences thereof; and communication with others about Internet information and its consequences. Questions were open-ended and revised iteratively, allowing for further exploration of new issues raised. For example, the topic of how to personally assess the quality and reliability of Internet information was introduced by participants and actively explored in subsequent interviews.

In the interviews, participants had the opportunity to extensively talk about their views, attitudes, and experiences. Probes according to the interview guide where used when the participants’ narratives came to an end or significantly deviated from the topic of interest. Interviews were conducted at a venue of the participants’ choice, which included a quiet room at the outpatient department, cafés, and people’s homes. Interviews were conducted by a researcher (author BS) who was not involved in the participants’ treatment. Interviews lasted 15 to 60 minutes. All were recorded on audiotape and transcribed verbatim. In addition, data were collected on a number of sociodemographic and illness-related variables.

### Data Analysis

For content analysis, QRS NVivo 7 software (QRS International Pty Ltd, Doncaster, Victoria, Australia) was used [27]. In all, 3 researchers (authors BS, IS, and MA) read the first 4 transcripts repeatedly to immerse themselves in the data. They independently separated the data into meaningful fragments identifying emerging themes and labeling them with descriptive codes. The individual coding frames were then compared and discussed until consensus was reached. BS and IS then applied the constant comparison method independently to chunks of 4 further transcripts at a time, applying and refining the coding frame by splitting broad themes into smaller fragments and merging smaller themes into broader categories as appropriate. The independent coding results were compared and discussed regularly, with BS applying the respective refined coding frame to the interviews that had been coded earlier. After 16 interviews had been coded in this way by BS and IS, MA independently coded another 4 interviews and discussed her findings with the other 2 researchers to validate the existing coding frame. All remaining interviews were then independently coded and compared by BS and IS, and ideas about themes and codings were discussed at regular intervals throughout the analysis. Recruitment, data collection, and analysis occurred simultaneously until theoretical saturation was reached.

### Specific Methodological Considerations

Repeated comparison and adaptation of the coding among researchers aimed to maximize the credibility of the results; that is, the fit between respondents’ views and the researchers’ reconstruction of the same. Dependability was ensured by a rigorous and traceable research process with all steps of the analysis being fully documented. Transferability is addressed in this report by providing background characteristics of the individual participants, confirmed by the provision of numerous verbatim quotes (see below), all of which contribute to the study’s validity and reliability [27-28].

## Results

### Characteristics of the Participants

Of the 26 participants whose data were required for theoretical saturation, 14 (54%) were male. The age range of all participants was between 18 and 52 years (mean 33). Sociodemographic characteristics of the participants are displayed in Table 1.

The majority of participants, (20/26 or 77%) reported their main diagnosis to be schizophrenia, while the remaining 23% (6/26) reported schizoaffective disorder. For all participants, the age at first onset of illness was between 11 and 44 years (mean 22). All but 2 participants had been hospitalized at least once. 

**Table 1:** Sociodemographic characteristics of the study participants

Characteristic		(n = 26)	%
**Gender**			
	Male	14	54
	Female	12	46
**Marital status**			
	Single	16	62
	Partnership/married	6	23
	Separated/divorced	4	15
**Employement status**			
	Unemployment benefit	4	15
	Social welfare benefit	1	4
	Student	3	12
	Disability pension	11	42
	Employed (including sick leave)	5	19
	other	2	8
**Living situation**			
	Own household (with partner/family)	9	35
	Own household (alone)	9	35
	Flat share	2	8
	Parents’ household	3	12
	Supported housing	3	12
**Education (highest level completed)**			
	No formal education	1	4
	Compulsory schooling^a^	5	19
	Primary education^a^	7	27
	Secondary education (including college and university)^b^	13	50

^a^ Different streams of basic education

^b^ Beginning at age 18 or 19

### Internet Use

General Internet use ranged from sporadic to several hours a day. Of all participants, 15 reported use of the Internet on a daily basis, 8 reported regular use, and 3 reported that they used the Internet only rarely. Of the 26 participants, 22 reported having searched for illness-related information on the Web. Of these, 5 regularly used the Internet for illness-related issues, while 17 did so occasionally. In addition, 14 had used chat rooms or networking sites; 5 had exchanged illness-related information there. Favored online sources were common search engines, Internet encyclopedias, and service-related websites.

### Thematic Analysis

We found that 7 key themes emerged from the data: (1) specific topics of interest on the Web, (2) reasons for and against using the Internet as a source of illness-related information, (3) subjectively perceived effects of information from the Internet, (4) communication with doctors about Internet content, (5) interaction about the illness on the Internet, (6) reliability and quality of Internet information, and (7) wishes and suggestions for improvement. All themes are outlined below together with some essential quotes. Additional quotes to support the results can be found in Multimedia Appendix 1. The emerging themes are summarized in Table 2 together with the number of participants talking about each topic and the total number of quotes coded within each category.

**Table 2:** Codes applied, number of people quoting the respective topic, and number of quotations within each category

Key Themes			Number of Participants	Number of Quotes
**Specific topics of interest on the Web**				
	Unspecific illness-related information		15	35
	Medication and side effects		14	43
	Diagnosis/symptomatology		14	18
	Services provided		13	21
	Risk factors and illness causes		6	10
	Prognosis and course of illness		5	6
**Reasons for and against using the Internet as a source of illness-related information**				
	Reasons for using the Internet for illness-related information		22	69
	Reasons against using the Internet for illness-related information		26	173
**Subjectively perceived effects of information from the Internet**				
	Positive effects		4	7
		Clarification and orientation	8	13
		Sharing	4	8
		Reassurance	6	7
		Finding one’s identity	3	4
	Negative effects		7	9
		Symptom provocation or aggravation	3	4
		Aversive emotional responses	12	25
	Effects on behaviors and attitudes		9	16
	Communication with doctors about Internet content		13	31
**Interaction about the illness on the Internet**				
	Interaction about the illness (nonspecific)		5	10
	Reasons for interaction		4	5
		Effects of interaction	4	9
	Reasons against interaction		17	28
	Reliability and quality of Internet information		23	69
	Wishes and suggestions for improvement		7	15

### Specific Topics of Interest on the Web

Frequently, interviewees reported having looked for general information with some association to their illness, and they often had problems defining the issues they had been interested in more precisely.

Medication was among the most frequently searched issues. Requests dealt with general information, for example, that lithium is a salt or the definition of generic drugs as well as personally relevant information such as side effects. The Internet was used to check whether side effects experienced were attributable to specific medication in the hope of finding better medication with fewer side effects.

Patient #26, male, age 35, schizophrenia since age 16 If somehow something new is on the market now again, if there is a new class that has no side effects at all...

Participants were conflicted between interest in the topic and the fear of finding out more about potential side effects. Many did not want to know too much about their medication and reported that they preferred relying on their doctors.

Patient #4, female, age 35, schizophrenia since age 10 And then I am afraid, because I don’t like to take medication that gives me cardiac death or apoplexy or stroke and there is a higher risk for diabetes as well.

Another important interest pertained to diagnostic criteria, symptomatic categories, and statistics, mainly to define one’s own illness and to verify one’s diagnosis.

Interviewees also talked about their Web searches for a variety of services, which they had conducted mainly to help them find a suitable facility or to better evaluate services before deciding to use them. Overall, they felt that such information reduced the barrier to actually accessing psychiatric help.

Patient #24, male, age 29, schizophrenia since age 2 I mainly wanted to know about the outpatient clinic, like what the opening hours are, when you can be admitted, or when you can speak to a doctor. So in the end, that’s exactly why I actually came here [to the outpatient department], because I found it fairly quickly [on the Internet]…here, it’s not really a problem to speak to a doctor.

When it comes to risk factors and illness causes, some potential psychosocial causes were mentioned (especially stress or predisposing personality factors), but, overall, biological illness models prevailed, especially drugs and assumed genetic and biochemical causes. These biological explanatory factors appeared to provide relief in dealing with the illness. Only a few participants remembered that they had read about prognosis and the likelihood of recovery, and, overall, such information was regarded as rather delicate.

### Reasons for Using the Internet to Find Illness-Related Information

Apart from general advantages such as easy access, speed, and the broad spectrum of information, illness-related motives for using the Internet also became apparent. These included the anonymity and absence of hierarchy on the Web, which we found to be associated with a lower perceived threshold to accessing information and with gaining confidence for overcoming problems with social interaction.

Patient #19, male, age 44, schizophrenia since age 23 Another advantage is that, that the Internet has a flat shape, that it is accessible to all of society.

Apart from positive incentives for Internet use such as anonymity and egalitarianism, negative incentives could also be identified, including dissatisfaction with therapy, problems communicating with the doctor, and the opportunity to find individually suitable answers.

Patient #4, female, age 35, schizophrenia since age 10 It’s also a source of information for people that are just starting on antipsychotics and who perhaps don’t get the information they want from their doctor, and it gives you the feeling that you got all the information.

### Reasons Against Using the Internet to Find Illness-Related Information

General reasons cited by interviewees against using the Internet to find illness-related information were lack of access to a computer, financial problems, difficulties using technology, fear of computer viruses, fear of Internet addiction, preference for other sources of information, and the expectation of low quality of Internet information. Further important reasons were that the demand for information had already been satisfied, lack of interest, and the wish to rely on a doctor.

Patient #25, male, age 26, schizophrenia since age 6 I don’t know, I somehow don’t believe that using the Internet can help. I trust my doctor.

The prominent illness-related reasons against Internet use were stimulus overflow and the inability to deal with the abundance of information, problems with concentration, lack of energy and depressive symptoms, paranoid ideas and fear of symptom provocation, and the wish to distance oneself from illness-related topics as part of the recovery process.

Patient #11, female, age 43, schizoaffective disorder since age 19 …that it is overstressing, that the inconsistencies within the information are strongest on the Internet…when you have too many opinions, you are lost in psychosis.

Patient #15, male, age 30, schizoaffective disorder since age 11 I try to get over that on my own, and I don’t want all this influence…I want to deal with things the way I want and not be blinded by some Internet report.

Patient #12, female, age 35, schizophrenia since age 12 And this is also a part of my illness, well, a part of my recovery to distance myself a little from that.

### Subjectively Perceived Effects of Information From the Internet

#### Positive Effects

Overall, positive effects may best be summarized as supporting empowerment by getting knowledge and improving access. Specifically, Internet information was considered to help patients better understand themselves and the illness, providing clarification and orientation.

Patient #18, male, age 26, schizophrenia since age 8 It [illness-related information on the Internet] has simply clarified a lot. And it was important for me to see how other people deal with it [schizophrenia] and how I could perhaps deal with it.

The possibility to anonymously tell one’s own story and to discover that other people reported similar experiences was perceived as a relief. This applied to direct exchange with others, for example, in chat rooms but also applied to the simple finding and reading of illness-related information.

Patient #20, male, age 52, schizoaffective disorder since age 22 …that I have the feeling not to be alone with the problems I have.

Information in itself had a reassuring effect, reducing fears (eg, of becoming addicted to medication) and helping to better integrate one’s situation and redefine one’s identity.

Patient #8, female, age 34, schizophrenia since age 5 The positive thing is, about the information, in principle, that you don’t get stuck in this “okay, now I’m nuts,” but that this is a disorder…there are biochemical causes and everything is quite simple in my opinion.

#### Negative Effects

Negative experiences were the provocation or aggravation of symptoms and aversive emotional responses, especially fear, sadness, and hopelessness, for example, in relation to dramatic illness stories.

Patient #9, female, age 46, schizoaffective disorder since age 26 You can get Internet-induced psychosis from that [searching for illness-related information on the Internet]; you can simply freak out.

Patient #8, female, age 34, schizophrenia since age 5 A negative effect was that you become scared, there is a lot more, ahem, there are not enough success stories on the Web, I’d say, and you get a lot of negative things.

#### Effects on Behavior and Attitudes

Internet information was found to have the potential to stimulate changes in behavior or attitudes. These were positive in most cases, for example, better coping strategies or lower thresholds for seeking help.

Patient #18, male, age 26, schizophrenia since age 8 To better cope with the illness and avoid situations, like drugs, for example, or excessive stress, or that you simply learn to take better care of yourself.

Negative effects on attitudes referred mainly to medication. Specifically, Internet information was described as leading to a more critical attitude toward one’s own medication.

Patient #11, female, age 43, schizoaffective disorder since age 19 If you read all that, you can’t take the meds anymore anyway, because they have more side effects than they have main effects.

### Communication With Doctors About Internet Information

Reasons not to talk to doctors about information from the Web were numerous. Among the most important fears were that doctors could feel criticized or have an unchangeable preconceived view and it wouldn’t be worth discussing things anyway.

Information from the Internet had the potential to significantly change the relationship with the attending doctors, with the most important aspect being a shift of the subjectively perceived hierarchy.

Patient #20, male, age 52, schizoaffective disorder since age 22 Well, that it is not such a downhill grade anymore, where he [the doctor] has the information about everything and I am there being fed by him and don’t really know why and what I am getting something for and what effects that can have and so on. This downhill grade is something that I have managed to level out, in terms of having power and becoming assertive as a patient.

The way doctors’ reactions were read by patients when talking about Internet information mainly depended on the quality of the patient-doctor relationship. In a good relationship, reactions were mostly judged as positive even when the doctor’s reply was evasive or even openly critical of the Internet search.

Patient #7, female, age 38, schizophrenia since 19 years Yes, she [the doctor] said there are so-called hypochondriacs who extensively surf the Web and imagine all kinds of illnesses and so on and that I should be careful with this kind of information. Yeah, and then I somehow thought she is right. I really don’t need to read all kinds of rubbish that doesn’t even affect me…

Occasionally patients felt left with uncertainty, doubt, and disappointment, for example, when the conversation did not lead to a satisfactory explanation or desired change. One interviewee recounted having found a new drug on the Internet not yet known to his doctor. While trying to avoid interpreting this incidence in the interview, the frustration it caused was obvious.

Patient #26, male, age 35, schizophrenia since age16 Yes, basically I am content with [my doctor], well, except for this one time, when he didn’t know that [the new medication]… if that is their specialist area they have to know about it, otherwise they can’t attend to patients.

### Interaction With Others About the Illness on the Internet

In all, 5 interviewees reported exchange of information with other people about the illness on the Web. All stressed the advantage of not being confronted with insecurities in personal contact and appreciated the special content of the information gathered in this way.

Patient #7, female, age 38, schizophrenia since age 19 People who really have someone in their family who has exactly the same illness, or who have it themselves, I think the things they can tell you are more interesting than when you ask the doctor.

The illness-related interaction on the Web was assessed positively throughout. Overall, 3 major effects were found: self-help or mutual help in coping with the illness, boosting self-esteem and self-validation through helping others, and reassurance through sharing one’s story.

Patient #18, male, age 26, schizophrenia since age 8 It is also about taking care of each other: How are you? How am I? What advice can I give you when you are unwell? Yes, and through that exchange you learn to deal with your illness and avoid situations that are bad, for example.

Participants who had never used the Internet for illness-related exchange talked about their reasons against it. Relevant obstacles were, again, problems with technology but also illness-related apprehensions, especially fear of becoming addicted, distrust of unknown people, and the necessity to protect oneself against other people’s illness stories.

Patient #7, female, age 38, schizophrenia since age 19 I think I would be very careful there, because I don’t know if that is a private person who’s logged in there, I mean, I don’t know if you can believe everything people write.

Patient #8, female, age 34, schizophrenia since 5 years Yes, there are also funny psychoses, but I think they are the minority, and most things people are experiencing are really dramatic, and I want to protect myself against that a little.

### Reliability and Quality of Internet Information

Information was described as interesting, rational, good, and credible but also as superficial, trivial, incorrect, of lesser quality, and bad, and even when information was perceived as satisfactory overall, often some skepticism remained about its quality and credibility.

Even though several interviewees had never thought about the reliability of Internet information, most were able to comment on potential strategies to assess its credibility. Often, the judgment was a personal, emotional, or intuitive decision that had to do with a “generally reputable impression” of a given Web page on which nothing should appear “strange” or that should not contain “flashy advertisements.” A further technique was to determine the provider, with more credibility being attributed to “official pages,” such as universities or magazines than to “private pages” or chat rooms.

Patient #20, male, age 52, schizoaffective disorder since age 22 You have to check the sender; I mean if that is a medical university, for example, or just someone and you don’t know who it is. I think that way you can differentiate very easily what is serious or professional and what isn’t.

Others evaluated the information according to its perceived comprehensibility, usefulness, and transparency. Comparison with one’s own experiences was another strategy, and congruency resulted in trust.

Patient #3, female, age 25, schizophrenia since age 9 …I have my own lived experience, and I can agree with that or not.

The appraisal by doctors, family, and friends also helped interviewees to form an opinion. Finally, technical features such as cookies, spyware, or the virus scan activation were mentioned as indicators of poor quality.

### Wishes and Suggestions for Improvement

Attributes of information wished for were that the information should be clear, objective, scientific, and actively destigmatizing. There was a demand for more reflected viewpoints of users and positive case histories, as well as more education about drugs such as cannabis.

Patient #7, female, age 38, schizophrenia since age 19 …you are most satisfied as a patient when you get really scientific explanations; that helps.

Patient #8, female, age 34, schizophrenia since age 5 [I would like] more positive case histories because actually you never read, for example, about people who managed to live completely normal lives again.

One of the wishes was for doctors to recommend good Internet sites and talk more about information obtained from the Web. Another suggestion was that doctors should explain on the Internet how they usually communicate with patients and that patients should be able to ask doctors specific questions directly on the Web.

## Discussion

### Parallels With the General Population

The participants in this study stated that they value the same advantages of the Internet that the general population values, notably, the easy and quick access, the broad spectrum of available information, and the anonymity of Internet use [3,29]. Strategies described for searching for information and assessing its quality also closely resembled those of the average Internet user [3]. Although the assessment of content was largely based on intuition and not restricted to specific indicators of quality, most interviewees reported personal strategies for assessing the quality and reliability of information. Similar to the general population, they were often concerned about the quality of content on the Internet, which, however, did not prevent them from using it [3,29].

Similarly, a number of arguments against Internet use for illness-related information that have been made by the general population were also made by the participants in the study. Among these were problems with technology or expectations of poor quality but also having sufficient information through other sources or preferences for direct personal information from professionals [3,30].

### Parallels With Other Patient Groups

While reasons against using the Internet were a prominent topic throughout the interviews and a lot of skepticism was expressed toward Internet contents, the Internet was still described as an influential source of illness-related information. Similar to patients with other diagnoses such as pain, interviewees reported that frequently sought information included diagnosis, medication, and specific services and that forums or chat sites were infrequently visited [31]. Retrieved information was perceived as helpful for better understanding oneself and the illness, as has been shown among people with poor health status in general [6,15]. Gaining the knowledge of not being the only person affected and anonymously learning how others deal with problems was a source of relief, and online health information in general led to a reduction of barriers to seeking professional help [5-6]. However, for the participants in our study, the retrieved information, especially dramatic illness stories, was also frequently perceived as disturbing, causing sadness, despair, and hopelessness or worsening the attitude toward medication.

As has been shown in studies involving people with somatic conditions [15,31-32], participants reported that they rarely spoke to their doctors about the results of their Internet searches, partly due to the fear that the doctors might feel criticized. However, Internet information increased participants’ confidence to talk to their physicians about concerns [15], and talking to their physicians about illness-related information from the Web facilitated an improvement in their relationships with their physicians [15,33]. For patients with schizophrenia in our study, a particularly important change was the perceived shift in the hierarchy to a more equal relationship. At the same time, a certain frustration and resignation concerning doctor-patient communication became apparent, especially when questions were not answered properly or did not result in a change of treatment.

### Psychosis Specific Issues

Among the specific illness-related reasons for using the Internet elucidated in this study were the anonymity and absence of hierarchy on the Internet, which has similarly been found among healthy individuals [29] but especially among people with chronic and stigmatizing conditions [5-6]. In this respect, a specific advantage for patients with psychosis, who often have pronounced fears and uncertainties in social interaction, was not having to face another person but still being able to gain information and interact with others without feeling devalued or unsafe. However, while a different study showed that people with schizotypal personality disorder specifically value information exchange connected with social interaction on the Web [16], our participants with schizophrenia attached higher importance to general information displayed on the Web and were rather skeptical about information from forums or chats. Another specific advantage for patients with schizophrenia was the opportunity to find idiosyncratic explanations and meaningful ways to express themselves in the context of the illness, for example, by accessing, producing, and combining pictures, music, and text from diverse sources.

Knowledge of specific illness-related problems or arguments against using the Internet among this patient group was one of the most significant findings of this study. Problems that were expressed included stimulus overflow and inability to deal with the abundance of information, difficulties with concentration during psychosis, lack of energy, paranoid ideas and fear of symptom provocation, and the necessity to distance oneself from illness-related topics as part of the recovery process. Participants also mentioned the possibility of an overabundance of information. It became evident that patients with schizophrenia may perceive only a certain amount of information as reasonable and feel the need to guard themselves against excess information. Overall, there was some ambivalence regarding the need for information and a struggle to achieve a subjectively adequate distance from illness-related topics and from other people with the same disorder and their stories.

### Limitations

The percentage of participants with secondary education shows that a large proportion of our sample was well educated. Given the diverse sample that was recruited not only from the university hospital but also from community psychiatrists and a low threshold community mental health organization, this may reflect the fact that among people with schizophrenia, those with higher education are more likely to use the Internet, as has also been shown for the general population and people with other disorders [8-9,15,34]. Moreover, people with higher education may simply be more inclined to participate in research.

Some of the participants referred to Internet use that had occurred long ago. This may have been especially true for those who were facing problems with the Internet leading to reduced Internet use. Hence, in these participants a memory bias may have impaired the recall of experiences with the Internet.

### Implications

The results of our study clearly show that the Internet is an important and influential source of information for patients with schizophrenia. Those who participated in our study wished for more communication with their doctors about information they have retrieved from the Internet. Research in different medical disciplines, however, shows that doctors only rarely integrate the Internet into their daily routine [4,35]. Such integration of the Internet into consultations was not only an explicit wish of the interviewees in our study, it also seems especially important for patients with schizophrenia given their specific problems with Internet use. Whether this increased wish for communication applies to patients with different background characteristics such as lower education remains to be investigated in larger quantitative studies.

Given the potential for change in health care utilization behavior or in attitudes toward treatments and doctors, patients’ Internet use may also have an indirect impact on mental health professionals which health professionals should be aware of in their general practice. Since this qualitative study revealed effects subjectively perceived by participants, the results can serve to generate informed hypotheses, while quantitative and prospective studies in particular are needed to empirically establish the potential effects of illness-related Internet use. Our study may also provide a basis for the development of a questionnaire as a foundation for the quantitative investigation of Internet use and its consequences among patients with schizophrenia.

Moreover, in recent years there has been an increasing tendency to use information technology, including the Internet, for patient education and therapeutic interventions for people with psychotic disorders [36-38]. In the design of such interventions, the specific problems, needs, and consequences of Internet use for people with schizophrenia should be carefully considered. Our study creates a first empirical basis to inform the continuous development of Internet-based interventions for this population.

## Multimedia Appendix 1

Additional quotes to support the study results

## Abbreviations

ICD-10: International Classification of Diseases, Tenth Revision
