# Metadata Correction: How Patients With Schizophrenia Use the Internet: Qualitative Study

**DOI:** 10.2196/jmir.3661

**Published:** 2014-07-10

**Authors:** Beate Schrank, Ingrid Sibitz, Annemarie Unger, Michaela Amering

**Affiliations:** ^1^Department of Psychiatry and PsychotherapyMedical University of ViennaViennaAustria

The authors of “How Patients With Schizophrenia Use the Internet: Qualitative Study” (J Med Internet Res 2010 Oct-Dec; 12(5): e70) have inadvertently declared on submission that all authors contributed equally. The author Schrank should have been listed as the sole first author, with the authors Sibitz, Unger, and Amering listed as co-authors, with adequate but not equal contributions. All authors have agreed to remove the “equal contribution” footnote (see Multimedia Appendix). Consequently, the “equal contribution” footnote has been removed in the corrected version of the paper on the JMIR website together with publishing this correction notice on July 30, 2014. A correction notice has been sent to PubMed and the correct full-text has been resubmitted to Pubmed Central and other full-text repositories.

## Multimedia Appendix 1

Statement signed by all authors agreeing to the corrigendum of authorship contributions.

