# Supplementary material for: How Patients With Schizophrenia Use the Internet: Qualitative Study
Source: J Med Internet Res. 2010 Dec 19;12(5):e70. doi: 10.2196/jmir.1550 (PMC3057320; doi:10.2196/jmir.1550)
Supplement: Supplementary file 1 [file jmir_v12i5e70_app1.pdf]

Online data supplement: additional quotes to support study findings

SPECIFIC TOPICS OF INTEREST ON THE WEB

Well, at the very beginning I knew practically nothing about psychiatric illnesses at all. So I tried to read up on things in general, what there is out there overall. [#8, female, age 34, schizophrenia since 5 years]

Medication & side effects

Well, yes, I simply realised that the symptoms were like mine, because I had had this presumption myself that it was due to Psychopax [Diazepam], and now, well that it was more severe with me, and then I realised that it is simply true what was written in there. [#15, male, age 30, schizoaffective disorder since 11 years]

I already went on a search for atypicals, for ones that don't have a weight issue. Ziprasidon is one like that for example, the only thing was that I got side effects in the ECG, so I got cardiac arrhythmia, so bad, that only happens to one in thousand patients. [#4, female, age 35, schizophrenia since 10 years]

No, medication not at all, 'cause there I have no idea what I could get. So for me it was simply important that I get a first appointment and that the prescription, it was actually about the prescription, that I can rely on the doctor and that I must be able to rely on him. [#24, male, age 29, schizophrenia since 2 years]

Diagnosis / symptomatology

I looked on the internet because of the tablets and how schizophrenia affects men, or psychosis, how it is for women, these kinds of statistics. [#3, female, age 25, schizophrenia since 9 years]

What is psychosis, for example. Because at the very beginning, when I had only been with my psychiatrist for a short time, when he gave me a prescription or referral, he always wrote schizoaffective psychosis as a diagnosis, but for some time now he doesn't write psychosis any more, but disorder instead. And somehow I was very interested in that, why, what psychosis actually is. [#20, male, age 52, schizoaffective disorder since 22 years]

Sure, you can find that on almost every page, Bleuler's 4 A's, or Schneider's 4 A's […] and productive and negative symptoms. [#4, female, age 35, schizophrenia since 10 years]

Services provided

Where the internet has helped me a lot is finding a therapist or a place to go. [#8, female, age 34, schizophrenia since 5 years]

It's like often that the clinics have a focus on something, so that they for example have a far-east religious touch or are anthroposophical. And I think that that is especially important in this area, what kind of a picture you have of humanity or if you really want to be so rigid, and rigidly scientific medical and according to the textbook, or if interpersonal stuff also plays a role, for example. [#8, female, age 34, schizophrenia since 5 years]

Where the internet address is listed I often look at the homepage as well, cause there it says, what they've specialized in or what they offer or, and that is also often interesting to read. [#1, female, age 52, schizophrenia since 8 years]

Risk factors & illness causes

That drugs play a role, as it was for me, I used to smoke up a lot, yes and that, very much so, and did a lot of counterproductive stuff as well, but I developed many mechanisms, so I wouldn't have to stay in reality, to avoid problems, yes, and in retrospect it then became clear to me, what I did wrong in many areas [#18, male, age 26, schizophrenia since 8 years]

That is the latest information I actually found, that it is a genetically determined illness, that maybe soon they'll find something that can be done against it, or with this gene-medication someone will find something at some point. [#4, female, age 35, schizophrenia since 10 years]

Prognosis & course of illness

Well, mainly I wanted to know what it means to have an illness like that, and what the prognosis is and whether there is cure. [#18, male, age 26, schizophrenia since 8 years]

There are not enough success stories on the internet […] I don't want to hear, that there are courses of illness that become increasingly dramatic, sure there are ones, but I just don't want to hear about it and uhm, it's like I want limited information. [#8, female, age 34, schizophrenia since 5 years]

REASONS FOR AND AGAINST USING THE INTERNET AS A SOURCE OF ILLNESS RELATED INFORMATION

Reasons for using the internet for illness related information

The difference is not that great, the same stuff comes up in conversations, the only difference is that there is a difference in scope. The internet definitely has a much broader scope on things [# 19, male, age 44, schizophrenia since 23 years]

So and that was really my major weakness back then, so that I, even like today am still totally isolated, and I was totally scared of meeting up with anyone or stuff like that. [#22, male, age 22, schizophrenia since 9 years]
People who are dissatisfied with the therapy of psychoses altogether, mainly of psychoses, that they have used the internet [# 19, male, age 44, schizophrenia since 23 years]

With doctors I realized, that you need to exactly, exactly know what you want to know, so you have to ask a very exact question and then keep digging, otherwise you'll never get an answer. [#7, female, age 38, schizophrenia since 19 years]

I did it [search the web] when I felt very bad, simply to read about the course of illness in other people. But I also did it to find expressions for these states, like pictures or artwork. I frequently reflect on my condition and with that in mind search the web. […] The positive thing is that there is room for personal expression. [#15, male, age 30, schizoaffective disorder since 11 years]

Reasons against using the internet for illness related information

That you get addicted, that you can easily tilt into it, that happens quickly, that you like it, or some of it, and then you have to do it all the time. [#3, female, age 25, schizophrenia since 9 years]

Yes, cause I already knew that from elsewhere. I either got it from books or seminars or other places, or from personal experience or something. [#11, female, age 43, schizoaffective disorder since 19 years]

Because so far I already received sufficient information about that from someone, from a doctor [#2, female, age 39, schizophrenia since 10 years]

I spend as little time as possible in front of the computer, cause that, cause the optical stuff really gets on my nerves, like having to sit there, and that makes me aggressive and it's too much, it's simply too much. [#12, female, age 35, schizophrenia since 12 years]

There are so many directions, that's very complex, and all the optical things you have to concentrate on, that's difficult to filter [#21, male, age 31, schizophrenia since 11 years]

Everything is becoming more and more and more and all that is too much for me somehow. [#12, female, age 35, schizophrenia since 12 years]

Na, not really, cause reading like that for hours, when there's a lot of text, is too exhausting for me [#1, female, age 52, schizophrenia since 8 years]

In order to use the internet you need concentration […] that's not always the case after psychosis. [# 19, male, age 44, schizophrenia since 23 years]

People who are depressed, it's like, they want to pull the sheets over their heads, and don't want to see any internet either. [#6, female, age 36, schizoaffective disorder since 16 years]

It's like, I know this guy for instance, who has a massive delusion of persecution, and he searched the internet a lot, and then he got a lot of problems because of it, mental ones, cause he simply knew too much, cause it says so much on the internet, too much knowledge is no good either. [#3, female, age 25, schizophrenia since 9 years]

Well, this focus on the illness can also be a bad thing. The good thing about psychosis is that there are times when you are free of it, moments where the psychotic consciousness takes a back seat, or is lost, or can be pushed aside. [# 19, male, age 44, schizophrenia since 23 years]

I don't want to give this area of knowledge so much space within my brain. [#9, female, age 46, schizoaffective disorder since 26 years]

SUBJECTIVELY PERCEIVED EFFECTS OF INFORMATION FROM THE INTERNET

Positive effects

I think this is very important. You have to understand yourself. I mean, everyone who is ill wants to know what he has got. [#7, female, age 38, schizophrenia since 19 years]

Well, cause it, it simply interested me also in the sense that it was my illness, a little bit from a medical point of view it also interested me, but mainly because it was my illness, in order to better to understand. [#13, male, age 35, schizophrenia since 11 years]

When you see that other people have the same thing then you don't feel so alone. [#10, female, age 18, schizophrenia since 7 years]

I simply read all these other people's personal stories and that brought huge relief, because at that time, somehow, I had a bit of a wrong picture of the world and the people, because I thought that everyone was better off than me. [#22, male, age 22, schizophrenia since 9 years]

I was very relieved, that for Dominal [Prothipendyl] there was this subordinate clause […] that you don't get addicted to it. [#22, male, age 22, schizophrenia since 9 years]

… because for the whole story, how I had behaved for five months, it was a relief somehow, that that fits in somewhere. [#14, male, age 23, schizophrenia since 6 years]

…and I found a really fitting description for that, for how I felt. [#22, male, age 22, schizophrenia since 9 years]

Negative effects

You rather easily get into manic states, when you just click about and search for things, that you get upset doing that. [#15, male, age 30, schizoaffective disorder since 11 years]

Back then it was a dangerous thing, yes, because dreams and reality started to intermix and then I was totally occupied with these things. [#18, male, age 26, schizophrenia since 8 years]

When these psychoses or all psychiatric illnesses can make you uncertain of yourself and of course answers that are too complicated can add to this insecurity. [# 19, male, age 44, schizophrenia since 23 years]

I was kind of alarmed. It's like, then you think, oh my god, what comes next! [#10, female, age 18, schizophrenia since 7 years]

What I found there was very scary. Especially when I read about suicide, and then I got that video of a man who shot himself into the head. I mean, that was a real video! [#14, male, age 23, schizophrenia since 6 years]

.. it makes you scared, it makes you sad and it makes you desperate. [#8, female, age 34, schizophrenia since 5 years]

People have no prior knowledge and can't integrate the information, I mean over time you become an expert, or at least you know the stuff better, but in the beginning that can be very hopeless [#4, female, age 35, schizophrenia since 10 years]

When you read, for example, that that one person slept at night and the he woke up and heard voices that he should actually kill his parents and things like that. That's shocking, of course! [#1, female, age 52, schizophrenia since 8 years]

Effects on behaviours and attitudes

It absolutely takes away that initial obstacle because you know what is written about it and how it is dealt with, that takes away the barrier to go there. [#8, female, age 34, schizophrenia since 5 years]

It then became clear to me, what I did wrong in many areas and I stopped taking drugs, I've been clean for three years now. [#18, male, age 26, schizophrenia since 8 years]

It can contribute to lowering the threshold a bit that you go to a psychiatrist at all. [#5, female, age 49, schizoaffective disorder since 27 years]

You are more critical towards your medication. [#4, female, age 35, schizophrenia since 10 years]

I saw what can happen, when you hear what dangers these drugs hold, side effects in any case and long-term damage, and then I saw what happened to some of the people I was friends with, as they became complete pill opponents, they didn't take any more medication and, uhm, basically just turned into these complete vegetables, any type of social communication like I had been used to before, was no longer possible after that. [#5, female, age 49, schizoaffective disorder since 27 years]

COMMUNICATION WITH DOCTORS ABOUT INTERNET CONTENT

As a patient you have practically no influence on what therapy you get and what medication you get, because that is very quickly interpreted as resistance or that you don't want to accept your disorder. I didn't really make the best experiences there. [#8, female, age 34, schizophrenia since 5 years]

How was that, to talk about that with your doctor? (BS)
Well, scary I mean, I am not afraid of my doctor, but everything that goes into the direction of critique, or own suggestions and so on, I am not so bold when it comes to that. [#20, male, age 52, schizoaffective disorder since 22 years]

Do you think you can talk about these things with doctors? (BS)
That's difficult. They have precast views about things. The effect is, everyone has to represent their discipline and many doctors are biased in many aspects of their opinion. [#16, male, age 23, schizophrenia since 2 years]

His reaction was basically rather positive. He said it is good when you try to inform yourself about your disorder. He didn't, not negatively, he just said that I should be careful not to be drawn into things again, which may be detrimental for me […] I can take that quite well, because I am not interested in having another episode again. [#18, male, age 26, schizophrenia since 8 years]

INTERACTION ABOUT THE ILLNESS ON THE INTERNET

Reasons for and against interaction

Body language becomes irrelevant, that's for sure, […] I am a person, who simply has this constant feeling that everything is pointless anyway, […] so if I don't want to sit in a corner by

myself, if I want to talk to someone, they can tell by looking at me, that I look like I don't really care, and that burdens me, and of course, on the internet you can just express your thoughts without having to worry about stuff like that. [#22, male, age 22, schizophrenia since 9 years]

Chatting makes you sick and addicted. Yes, I know people who started to chat and then where unable to stop again. [#3, female, age 25, schizophrenia since 9 years]

You never really know who the person sitting on the other side really is. [#10, female, age 18, schizophrenia since 7 years]

I don't know, someone who you otherwise would never talk to or someone who draws you into something, even if you don't believe it and don't think about it. These stories psychotic people tell you leave their traces in one's own judgement, one's own sensation, one's own perception. It's very difficult to listen to someone with a psychosis when you have a psychosis yourself. [#4, female, age 35, schizophrenia since 10 years]

Very often when talking to other affected people there is a lot of panic and helplessness and aggression, and on the other hand I know that many of the affected want to convert others. I have heard that, and I don't want to hear it any more. [# 19, male, age 44, schizophrenia since 23 years]

Effects of interaction

He was there, but I was there for him as well, so that was built on reciprocity and he doesn't have a lot of experience with psychotherapy or stuff like that either, but we have simply helped each other. [#22, male, age 22, schizophrenia since 9 years]

It has helped me, because it is a good feeling when you can help someone, when you can give advice. [#18, male, age 26, schizophrenia since 8 years]

Then I published my personal story, that was quite long, and quite a lot of people contacted me then and sent private messages, not public replies but direct messages to me and in my case that was a stroke of luck, a guy from Munich got in touch and really built up my confidence in that time. [#22, male, age 22, schizophrenia since 9 years]

RELIABILITY AND QUALITY OF INTERNET INFORMATION

I can't really tell, it has got a lot to do with feeling. [#18, male, age 26, schizophrenia since 8 years]

I didn't judge it at all. I took as granted, what was written there. [#4, female, age 35, schizophrenia since 10 years]

I don't know, I simply realised when friends talk about it and they say there is a lexicon or another thing, National Geographic for example, then I simply trust in these things. [#18, male, age 26, schizophrenia since 8 years]

First, people told me if you want to know something look it up on Wikipedia, but then I found out that anyone can write something into Wikipedia, and no one controls it or challenges that. I mean, where is the reliability of such a medium? [#5, female, age 49, schizoaffective disorder since 27 years]

And when do you perceive something as strange? (BS)

Mainly when I don't really understand it. [#1, female, age 52, schizophrenia since 8 years]
And when don't you understand something? (BS)
Well, probably when there is too much information, or when I find something I am not really interested in and therefore not understand it. [#1, female, age 52, schizophrenia since 8 years]

And what kind of information did you find? (BS)
Good information. [#17]
What kind of information is good information? (BS)
Well, when it's useful. [#17, male, age 24, schizophrenia since 4 years]

Then I consult with my daughter or my husband when I find there is anything strange about something. [#1, female, age 52, schizophrenia since 8 years]

For wrong information, you just have to check if there is some advertisement again. [#26, male, age 35, schizophrenia since 16 years]

There are certain pages where the virus scan is automatically activated and says I shouldn't open that page. Then I don't open it. [#18, male, age 26, schizophrenia since 8 years]

In the source code. In HTML it's possible to check the source code of a page and there you can see if there are cookies or spyware or the resolution of the header and how it is written, and thereby you can check out the page. [#16, male, age 23, schizophrenia since 2 years]

WHISHES AND SUGGESTIONS FOR IMPROVEMENT

Demands and suggestions for improvement of the internet

It would be good to have a page with information on overlapping illnesses, where they also say that Neuroleptics can be used for different disorders to take away this immediate connection with schizophrenia. Especially for patients with schizophrenia that would be an advantage, not so much stigma, not on the medication and not on the disorder. [#4, female, age 35, schizophrenia since 10 years]

It would be good if there was a page where people who are affected by this can write something. Of course the danger there again is that only those people write something who are opposed to things. Because those are always the first and they are usually the loudest and that is also dangerous because it is stigmatising for all the satisfied users of psychiatry. I'd say those who are reflected about themselves should have the word. [#5, female, age 49, schizoaffective disorder since 27 years]

Another thing I think should be on the internet is more on the effects that drugs can have, that drugs can cause schizophrenia, on sites dealing with drugs or so, a lot more than there is now. [#4, female, age 35, schizophrenia since 10 years]

Demands for improvement directed at doctors

I would advise doctors to carry a small table with useful internet addresses with them, addresses that the doctor thinks would be recommendable that one should have a brief look at. [#4, female, age 35, schizophrenia since 10 years]

I do believe, that a page on the internet where doctors could present something they want, like something they would normally tell patients on the ward, or the stuff they tell people in their private practice, if they put that on the internet, then I think that that could contribute to lowering the threshold to even considering going to see a psychiatrist. [#5, female, age 49, schizoaffective disorder since 27 years]

For example, the combination of one drug with another which you possibly tolerate better, that would be an interesting question which you normally can't ask. Or is there any kind of institution on the web where you can pose questions like that? [#6, female, age 36, schizoaffective disorder since 16 years]
