# Supplementary material for: Metadata Correction: How Patients With Schizophrenia Use the Internet: Qualitative Study
Source: J Med Internet Res. 2014 Jul 10;16(7):e165. doi: 10.2196/jmir.3661 (PMC4115654; doi:10.2196/jmir.3661)
Supplement: Supplementary file 1 [file jmir_v16i7e165_app1.pdf]

To the *Journal of Medical Internet Research*
Editor and publisher: Prof. Gunther Eysenbach
geysenba@uhnres.utoronto.ca

Vienna, 30 June 2014

Re: **Corrigendum for "How Patients With Schizophrenia Use the Internet: Qualitative Study." J Med Internet Res 2010 Oct-Dec; 12(5): e70, doi: 10.2196/jmir.1550**

Dear Editor,

We have realised that all authors listed on the above publication are described as equally contributing authors. We herewith want to clarify that this description is not correct.

While all co-authors have contributed in a way that justifies their listing as co-authors, contributions were not equal. We declare that Beate Schrank should be listed as the sole first author and all remaining authors as co-authors only.

We appreciate that this may be slightly unusual more than three years after publication, but we only now realised the mistake. We request to correct this mistake and change the authorship to a single first author and three co-authors.

All authors agree to the authorship change and approve of the corrigendum as submitted.

With kind regards,

Beate Schrank[1]
beate.schrank@meduniwien.ac.at

Ingrid Sibitz[1]
ingrid.sibitz@meduniwien.ac.at

Annemarie Unger[1]
annemarie.unger@meduniwien.ac.at

Michaela Amering[1]
michaela.amering@meduniwien.ac.at

[1] Department of Psychiatry and Psychotherapy, Medical University of Vienna, Waehringerguertel 18-20, Vienna, 1090, Austria
